# Inherent differences of small airway contraction and Ca^2+^ oscillations in airway smooth muscle cells between BALB/c and C57BL/6 mouse strains

**DOI:** 10.3389/fcell.2023.1202573

**Published:** 2023-06-05

**Authors:** Zijian Zeng, Mengxin Cheng, Meng Li, Tao Wang, Fuqiang Wen, Michael J. Sanderson, James Sneyd, Yongchun Shen, Jun Chen

**Affiliations:** ^1^ Department of Pulmonary and Critical Care Medicine, West China Hospital, Sichuan University and Division of Pulmonary Diseases, State Key Laboratory of Biotherapy, Chengdu, Sichuan, China; ^2^ Department of Microbiology and Physiological Systems, University of Massachusetts Medical School, Worcester, MA, United States; ^3^ Department of Mathematics, The University of Auckland, Auckland, New Zealand

**Keywords:** airway smooth muscle, asthma, precision-cut lung slices, Ca^2+^ oscillations, airway hyperresponsiveness

## Abstract

BALB/c and C57BL/6 mouse strains are widely used as animal model in studies of respiratory diseases, such as asthma. Asthma is characterized by airway hyperresponsiveness, which is eventually resulted from the excessive airway smooth muscle (ASM) contraction mediated by Ca^2+^ oscillations in ASM cells. It is reported that BALB/c mice have inherently higher airway responsiveness, but show no different contractive response of tracheal ring as compared to C57BL/6 mice. However, whether the different airway responsiveness is due to the different extents of small airway contraction, and what’s underlying mechanism remains unknown. Here, we assess agonist-induced small airway contraction and Ca^2+^ oscillations in ASM cells between BALB/c and C57BL/6 mice by using precision-cut lung slices (PCLS). We found that BALB/c mice showed an intrinsically stronger extent of small airway narrowing and faster Ca^2+^ oscillations in ASM cells in response to agonists. These differences were associated with a higher magnitude of Ca^2+^ influx via store-operated Ca^2+^ entry (SOCE), as a result of increased expression of SOCE components (STIM1, Orai1) in the ASM cells of small airway of BALB/c mice. An established mathematical model and experimental results suggested that the increased SOC current could result in increased agonist-induced Ca^2+^ oscillations. Therefore, the inherently higher SOC underlies the increased Ca^2+^ oscillation frequency in ASM cells and stronger small airway contraction in BALB/c mice, thus higher airway responsiveness in BALB/c than C57BL/6 mouse strain.

## 1 Introduction

Asthma is clinically characterized by airway hyperresponsiveness (AHR), a phenomenon of excessive airway narrowing in response to agonists. Although AHR might result from hypertrophy and hyperplasia of airway smooth muscle (ASM) induced by airway inflammation (Bentley and Hershenson, 2008), the contractility of ASM itself also contributes to AHR. ASM contraction is driven by the phosphorylation of myosin light-chain (MLC) via the activation of MLC kinase, which is induced by the binding of calmodulin and intracellular calcium (Sieck et al., 2001). Previous reports have shown that agonist-induced ASM contraction is accompanied by repetitive waves of increased intracellular calcium concentration ([Ca^2+^]_i_) of ASM, termed Ca^2+^ oscillations, the frequency of Ca^2+^ oscillations determines the extent of ASM contraction (Prakash et al., 1998; Perez and Sanderson, 2005). Notably, the sustained Ca^2+^ oscillations cause a small and continual loss of Ca^2+^ across the plasma membrane (PM) via PM Ca^2+^ ATPases (PMCA) and Na^+^/Ca^2+^ exchanger (NCX). Therefore, the maintenance of agonist-induced Ca^2+^ oscillations and ASM contraction requires an influx of extracellular Ca^2+^. We has demonstrated that this influx occurs mainly via store-operated calcium entry (SOCE) (Chen and Sanderson, 2017), which involves a PM-resident Ca^2+^ channel called Orai1, and a sarcoplasmic reticulum (SR) membrane-spanning Ca^2+^-sensing protein called stromal interaction molecule 1 (STIM1) (Prakriya and Lewis, 2015).

Mouse is commonly proposed as an experimental animal model to investigate the pathogenesis of AHR. Different mouse strains were used to measure airway reactivity, therefore, the inherent differences among mouse strains have been observed. For example, two widely used mouse strains, BALB/c and C57BL/6, have been demonstrated to display substantial differences in the distribution of inflammatory cells, expression of proinflammatory cytokines, ASM mass, and subepithelial collagen and fibronectin deposition after acute or chronic exposure to allergens (Gueders et al., 2009; Van Hove et al., 2009). It is worth noting that BALB/c mice showed stronger agonist-induced airway responsiveness and increased shortening velocity of ASM than C57BL/6 mice (Duguet et al., 2000). However, BALB/c and C57BL/6 mice show no difference in the contractive response of tracheal ring segments to agonists (Safholm et al., 2011), indicating that tracheal contraction might not account for the different airway responsiveness. Recently, small airway contraction has been increasingly acknowledged in the pathogenies of AHR (Kjellberg et al., 2016; Stenberg et al., 2017). Nevertheless, it is still unclear whether BALB/c and C57BL/6 mice have inherent differences in the small airway contractility and whether the Ca^2+^ oscillations regulated by the magnitude of SOCE contributes to these differences.

In this study, we used an established precision-cut lung slices (PCLS) to access the agonist-induced contraction of the small airway and Ca^2+^ oscillations of ASM cells in BALB/c and C57BL/6 mice. We report that BALB/c mice show an inherently stronger extent of small airway narrowing and a higher frequency of Ca^2+^ oscillation in ASM cells in response to agonists, which might be associated with an increased Ca^2+^ influx via SOCE.

## 2 Methods and materials

### 2.1 Animals

Age matched female C57BL/6 mice and BALB/c mice (8–12 weeks with bodyweight of 19–22 g) were purchased from Charles River Breeding Laboratories (Needham, MA, United States) and GemPharmatech Co. Ltd (Nanjing, Jiangsu, China). The mice were bred in a specific pathogen free environment. The adequate number of mice was estimated by a power analysis. All protocols were in accordance with the Guide for the Care and Use of Laboratory Animals and approved by the Institutional Animal Care and Use Committee of University of Massachusetts Medical School and West China Hospital (approval number: 2018049A).

### 2.2 Reagents

Hanks’ Balanced Salt Solution (HBSS) was prepared by supplementing 20 mM HEPES buffer and adjusted to pH 7.4 (sHBSS). Hanks’ 0-Ca^2+^ solution (0-Ca^2+^ sHBSS) was prepared with no-Ca^2+^ HBSS supplemented with 1 mM Na_2_H_2_-EGTA, 20 mM HEPES and 0.9 mM MgSO_4_. Oregon Green 488 BAPTA-1 a.m. was purchased from Life Technologies (Grand Island, NY, United States). Ryanodine was purchased from Abcam Inc (Cambridge, MA, United States), other reagents were purchased from either Sigma-Aldrich (St. Louis, MO, United States) or Thermo Fisher Scientific (Pittsburgh, PA, United States).

### 2.3 Precision-cut lung slices (PCLS)

The detailed protocol has been previously described (Perez and Sanderson, 2005). 1.8% low melting point agarose was prepared in sHBSS. Mice were killed by cervical dislocation, and the lung was inflated with ∼1 mL 1.8% low melting point agarose via an intratracheal catheter at 37°C, 0.3 mL air was injected subsequently to flush the agarose within the airway into the distal alveoli. The cardiac lobe was disassembled after gelling agarose with 4°C sHBSS, and sectioned into 180 μm thick PCLS by a vibratome (VF-300, Precisionary Instruments, Greenville, NC, United States). As the slicing might stimulate a spontaneous airway contraction in PCLS preparation, all experiments were performed on the second day of slicing when the airways were fully relaxed. The PCLS were maintained in DMEM supplemented with antibiotics at 10% CO_2_ and 37°C for up to 2 days, within the periods the contractility of small airway remains unchanged. The experiments were performed at 37°C, and repeated at room temperature, in a custom-made, temperature-controlled microscope enclosure as described previously (Bai and Sanderson, 2009).

### 2.4 Airway contraction and relaxation

The detailed protocol has been previously described (Chen and Sanderson, 2017). The PCLS were held down by a 200 μm nylon mesh with a hole aligned over a selected airway on a cover-glass. To make a perfusion chamber, a smaller cover-glass was placed on the top of the nylon mesh and the side edges were sealed with silicone grease. We used two different agonists, methacholine (MCh) and 5-hydroxytryptamine (5-HT), which bind to their respective cell-surface receptors and activate different downstream G protein-coupled pathways to trigger airway contraction.

The contraction assay was performed by giving increasing doses (100, 200, 400, 800, 2000 nM) of MCh/5-HT sequentially with washing-out of agonists by sHBSS between each dose. The extent of airway narrowing in response to the agonists was determined by the change in lumen area, which was recorded by a CCD camera at 1 image per 2 s on an inverted microscope (Nikon Diaphot or Olympus IX71). The area of the lumen was calculated by using ImageJ, and then normalized by the formula: Area (%) = 100% * Lumen area at a certain time/Initial lumen area.

To compare the extent of airway narrowing between BALB/c and C57BL/6 mice, the extent of airway contraction was defined and calculated by the formula: airway contraction (%) = 100% *(Initial lumen area-lumen area at a certain time)/Initial lumen area. Then a dose-response curve was plotted by using the extent of airway contraction in response to increasing doses of agonists at sequential timepoints (5, 15, 25, 35 and 45 min) of the contraction assay. For each assay, 6 BALB/c mice and 6 C57BL/6 mice were used, 1–2 PCLS were obtained from each mouse, 2 randomly selected airways in each PCLS were assessed, and specific sample sizes were reported in the figure legends.

### 2.5 Intracellular Ca^2+^ signaling

The detailed protocol has been previously described (Chen and Sanderson, 2017). The PCLS were loaded with 20 μM Oregon Green (OG) 488 BAPTA-1-AM supplemented with 0.1% Pluronic F-127 and 200 μM sulfobromophthalein (SB) at 30°C for 1 h, and then placed in 200 μM SB at room temperature for 30 min to allow the de-esterification of OG. After washing the PCLS with sHBSS in the perfusion chamber, the intracellular Ca^2+^ signaling of a single ASM cell in the PCLS was recorded by a custom-built, video-rate scanning 2-photon laser microscope at a rate of 15 images per second. The change in fluorescence intensity in an interest region (8 × 10 pixels) of an ASM cell was analyzed by Video Savant 4 (IO Industries, Montreal, Canada) with custom-written code. The fluorescence intensity at a particular time (F_t_) was normalized to the initial time (F_0_) as F_t_/F_0_. For each assay, five to six BALB/c mice and five to six C57BL/6 mice were used, 1–2 PCLS were obtained from each mouse, two to three randomly selected ASM cells in each PCLS were assessed, and specific sample sizes were reported in the figure legends.

### 2.6 SOCE-activated PCLS

The validation of SOCE-activated PCLS has been reported (Bai and Sanderson, 2006). Briefly, the PCLS was treated with 20 mM caffeine and 50 μM ryanodine simultaneously for 5 min, followed by a thorough washout with sHBSS. This treatment gates the ryanodine receptors (RyR) of SR in an open state and leads to a decreased Ca^2+^ concentration in SR ([Ca^2+^]_SR_), activating SOCE to induce Ca^2+^ influx. The SOCE-activated PCLS remains viable for at least 5 h, during this time the [Ca^2+^]_SR_ is kept in a low level due to the persistent opening of RyR, and consequently the activation of SOCE is sustained and irreversible. As a result, the [Ca^2+^]_i_ is determined by the extracellular Ca^2+^ concentration ([Ca^2+^]_e_) via SOC influx.

### 2.7 Immunofluorescence

Mice were anesthetized with intraperitoneal injection of 2.5% sodium pentobarbital, and subsequently euthanized through exsanguination from the right ventricle to collect lung tissue. The left lung was inflated and preserved with 10% formaldehyde solution, followed by paraffin embedding and slicing. We employed tyramide signal amplification (TSA) principle for immunofluorescence staining, by using a multiplex fluorescence kit and following the guidelines provided by the manufacturer (RS0035, Immunoway). Briefly, the lung sections underwent dewaxing and rehydration, followed by antigen retrieval. Protein blocking was performed using peroxidase blocking solution for 30 min before each of the three staining cycles. Primary antibodies against Na^+^/K^+^ ATPase (NKA, 1:200, YT2973, Immunoway), α-smooth muscle actin (α-SMA, 1:200, 19245S, CST), STIM1 (1:200, ET1612-53, Huabio), and Orai1 (1:200, ER 1803–11, Huabio) were applied to the lung sections respectively for 2 h at room temperature. Then horseradish peroxidase (HRP)-labeled rabbit secondary antibodies were applied to the lung sections for 20 min, followed by incubation with TSA opal fluorophore (Opal 488, Opal 594 or Opal 647) for 10 min at room temperature. The antibody-TSA complex was removed through a heat-mediated antigen retrieval process using Tris/EDTA buffer (pH 9.0) during each staining cycle. Then the lung sections were counterstained with DAPI (P36931, Invitrogen). The immunofluorescence images were captured by Nikon A1R MP confocal microscope. We conducted a negative control to rule out the possibility of spontaneous fluorescence and non-specific signals by staining lung sections with vehicle instead of primary antibodies. The α-SMA (conjugated to Opal 488) was used to identify ASM cells. The fluorescence intensity of NKA (conjugated to Opal 594) was used as an internal control. We then quantified the fluorescence density of Orai1 and STIM1 (both conjugated to Opal 647) by normalizing the fluorescence intensity of Orai1/STIM1 to that of NKA in the same region of interest of ASM cells using ImageJ. Three BALB/c mice and three C57BL/6 mice were used for each staining, and two to three lung sections were obtained from each mouse. Three randomly selected ASM cells in each lung section were analyzed in a blinded manner, the specific sample sizes were reported in the figure legends.

### 2.8 Statistics

Data were statistically analyzed and plotted by GraphPad Prism 7.00 (GraphPad Software Inc., San Diego, CA, United States). For the comparison of two groups, the distribution of data was first tested by the Shapiro-Wilk normality test or KS normality test, and then the data were compared using the Unpaired *t*-test or Mann-Whitney test. For the comparison of dose-response curves, nonlinear regression analysis was performed first, and then the best-fits values, including the bottom, top and concentration for 50% of the maximal effect (EC50) of the curves, were compared between groups. Data are presented as the mean ± SD, the sample sizes and numbers of mice used in experiments were reported in the figure legends; a value of *p* < 0.05 was considered significant.

### 2.9 Mathematical model

The mathematical model has been reported previously and well validated in stimulating the dynamic structure of Ca^2+^ oscillations in the ASM of PCLS (Wang et al., 2010; Croisier et al., 2013; Croisier et al., 2015; Sneyd et al., 2017). In brief, the dynamics of intracellular Ca^2+^ concentration (c = [Ca^2+^]_i_) and the SR Ca^2+^ concentration (c_s_ = [Ca^2+^]_SR_) are modelled by:

$$\frac{dc}{dt}=J_{in}-J_{PMCA}+J_{rel}-J_{SERCA}$$

$$\frac{dc_{s}}{dt}=\gamma \left(J_{SERCA}-J_{rel}\right)$$

where *J*_*in*_ accounts for the Ca^2+^ influx, primarily through SOCE, while *J*_*PMCA*_ and *J*_*SERCA*_ represent the Ca^2+^ fluxes through the PMCA and SR Ca^2+^ ATPases (SERCA) pumps, respectively. *J*_*rel*_ *,* on the other hand, models the efflux of Ca^2+^ from the SR through inositol trisphosphate (IP_3_) receptors (IP_3_R) and RyR. The comprehensive description of the mathematical model is provided in the supplement.

## 3 Results

### 3.1 BALB/c mice showed stronger agonist-induced small airway contraction than C57BL/6 mice

To assess the extent of “small airway” narrowing, we distinguished airway generation as reported previously (Bai et al., 2007): the first airway generation was defined as the most distal airway that had an intact epithelium with ciliated cells, and then the next airway generation was identified when another airway branch merged into the airway. By sequentially cutting the PCLS from the cardiac lobe, 7–10 intrapulmonary generations of airways can be obtained. We defined “small airway” as airway generation four to five with a lumen diameter of approximately 0.2mm, which showed the strongest contraction compared to other airway generations (Bai et al., 2007). As the extent of small airway narrowing might be also affected by extracellular matrix tethering, which is mainly determined by the presence of agarose in the parenchymal spaces (Hiorns et al., 2016), we used an identical concentration of agarose (with identical stiffness after gelling) and carefully chose airways from similar positions in the same lobe of mice.

**FIGURE 1:**
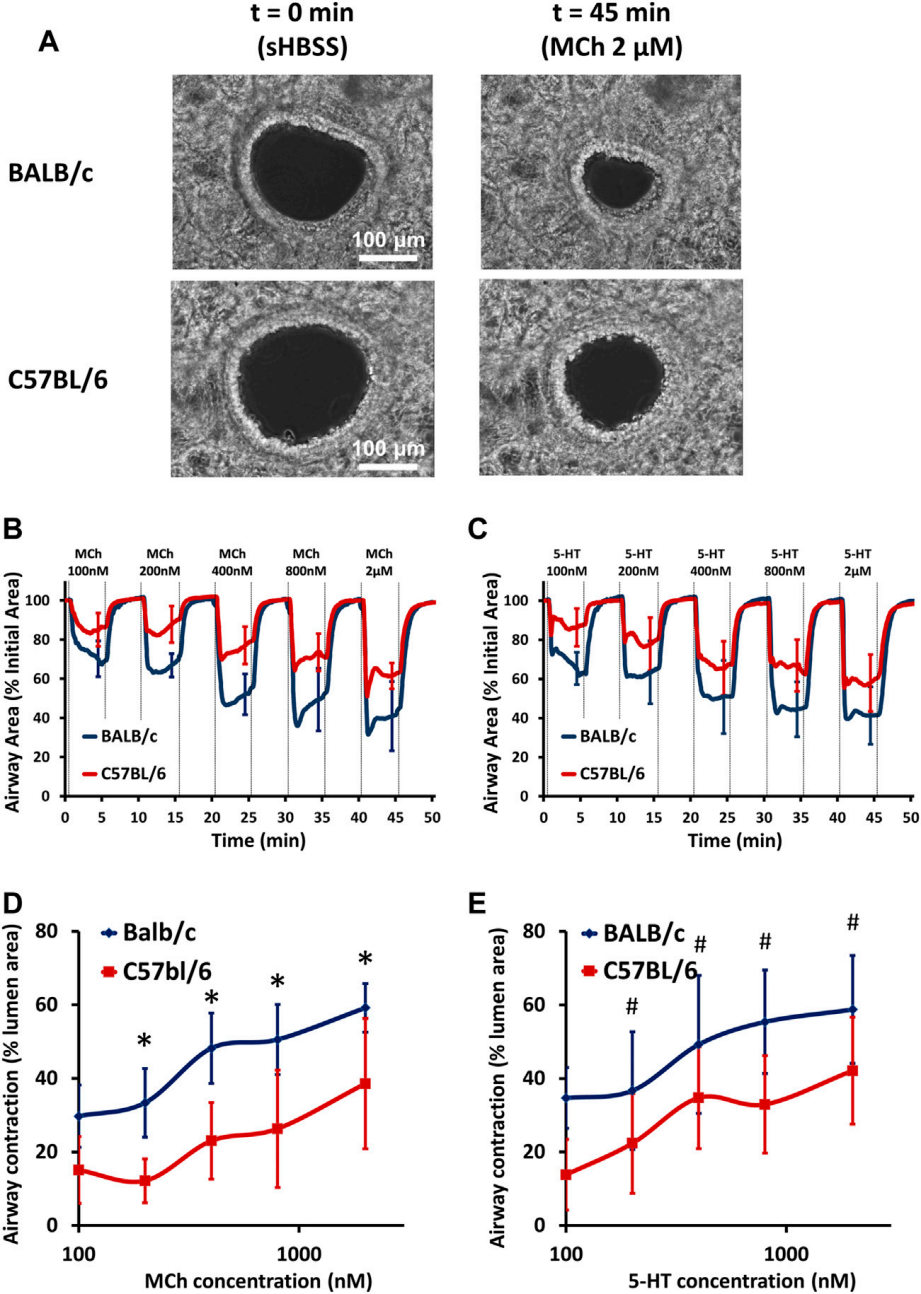
BALB/c mice show stronger airway contraction than C57BL/6 mice. **(A)** Representative phase-contrast images of 2 small airways in PCLS derived from BALB/c and C57BL/6 mice under resting conditions (left panel, scale bar = 100 μm) and exposed to 2 μM MCh (right panel) at 37°C. **(B–C)** The contraction assay show small airway contraction in response to increasing doses of MCh and 5-HT, respectively, at 37°C in PCLS derived from BALB/c (BLUE line) and C57BL/6 (RED line) mice. **(D–E)** The comparison of dose-response curves to MCh (D) and 5-HT (E) between BALB/c (BLUE line) and C57BL/6 (RED line) mice. Data are presented as the mean ± SD, *p < 0.0001 in (D), #*p* < 0.0001 in (E). Data are obtained from 14 airways in 8 PCLS of 6 BALB/c mice, and 12 airways in 6 PCLS of 6 C57BL/6 mice, respectively.

The extent of small airway narrowing in PCLS derived from BALB/c and C57BL/6 mice was visualized (Figure 1A) and analyzed (Figures 1B–E and Supplementary Figure S1) respectively. No pre-contraction of airways was observed in either BALB/c or C57BL/6 PCLS as previously reported (Chen et al., 2017). Both MCh (Figure 1B) and 5-HT (Figure 1C) induced dose-dependent small airway narrowing (as reflected by the change in airway lumen area) in the PCLS of BALB/c and C57BL/6 mice at 37°C. Compared to C57BL/6 mice, the extent of small airway narrowing in BALB/c mice was significantly stronger upon stimulation with both MCh (Figures 1B,D) and 5-HT (Figures 1C,E) at all concentrations tested (100–2000 nM), suggesting an overall increased small airway contraction in BALB/c mice. A similar pattern of increased airway contraction in BALB/c mice was also seen at room temperature (Supplementary Figure S1). These results demonstrate an intrinsically stronger small airway contraction in BALB/c mice than in C57BL/6 mice.

### 3.2 BALB/c mice showed higher Ca^2+^ oscillation frequency in ASM cells than C57BL/6 mice

**FIGURE 2:**
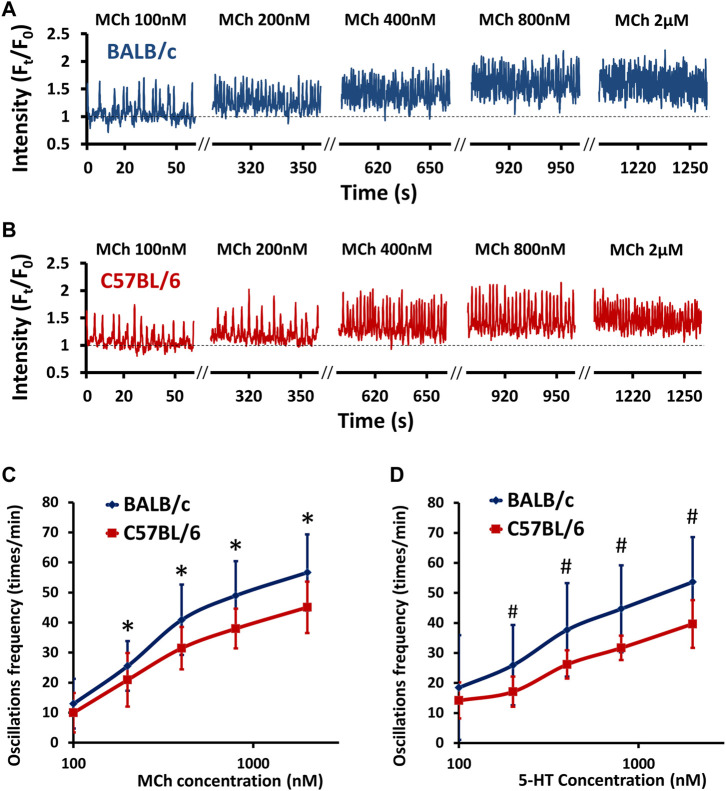
BALB/c mice show an increased agonist-induced Ca^2+^ oscillation frequency in ASM cells compared to C57BL/6 mice. **(A–B)** Representative traces show Ca^2+^ oscillations of ASM cells in PCLS derived from BALB/c (A, BLUE line) and C57BL/6 (B, RED line) mice in response to increasing doses of MCh at 37°C. **(C–D)** The comparison of dose-response curves to MCh (C) and 5-HT (D) between BALB/c (BLUE line) and C57BL/6 (RED line) mice. Data are presented as the mean ± SD, *p < 0.0001 in (C), #*p* < 0.0001 in (D). Data are obtained from 18 ASM cells in 9 PCLS of 6 BALB/c mice, and15 ASM cells in 7 PCLS of 6 C57BL/6 mice, respectively.

Agonist-induced small airway contraction is largely induced by the contraction of ASM itself, which is mediated by intracellular Ca^2+^ oscillations (Perez and Sanderson, 2005). Therefore, we examined the intrinsic difference in Ca^2+^ oscillations of ASM cells stimulated by MCh and 5-HT. No spontaneous Ca^2+^ signaling was observed in ASM cells derived from either BALB/c or C57BL/6 PCLS as previously reported (Chen et al., 2017). In the presence of both MCh and 5-HT at 37°C, ASM cells exhibited periodic Ca^2+^ waves propagating along the length of the cells (Figures 2A,B), accompanied by ASM contraction. In the ASM cells of both BALB/c and C57BL/6 mice, MCh induced a dose-dependent increase in Ca^2+^ oscillation frequency (Figures 2A,B). Compared to C57BL/6 mice, the ASM cells derived from BALB/c mice showed significantly increased Ca^2+^ oscillation frequency upon stimulation with both MCh (Figures 2A–C) and 5-HT (Figure 2D) at all concentrations tested (100–2000 nM). A similar pattern of higher Ca^2+^ oscillation frequency in ASM cells of BALB/c mice was also noted at room temperature (Supplementary Figure S2). These results demonstrate an intrinsically higher Ca^2+^ oscillation frequency in ASM cells of BALB/c mice than in those of C57BL/6 mice.

### 3.3 The frequency of agonist-induced Ca^2+^ oscillation depends on the magnitude of SOCE

**FIGURE 3:**
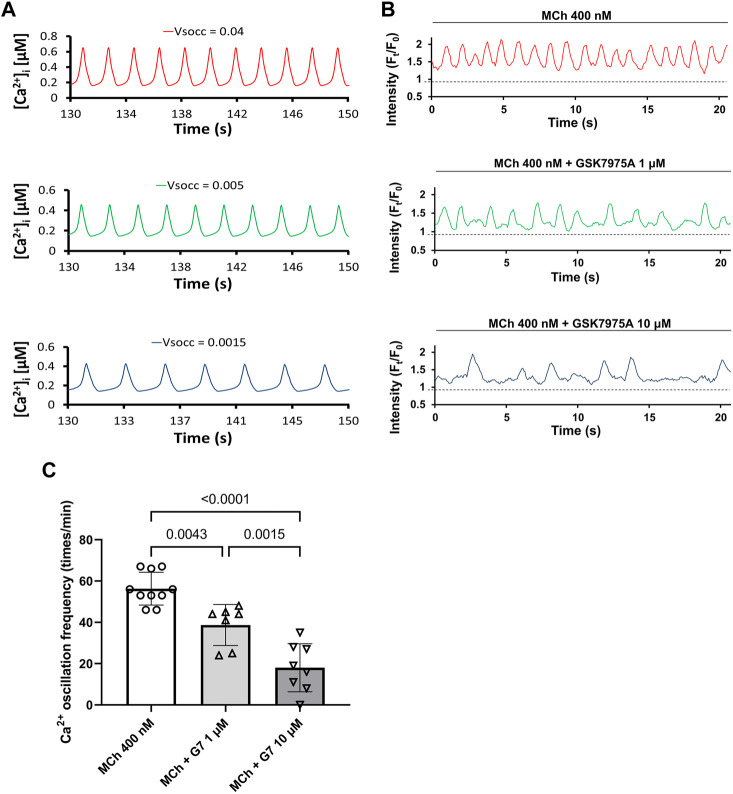
Reduced SOC current (V_SOCC_) leads to slower Ca^2+^ oscillations in ASM cells. **(A)** Mathematical model predicts that decreased V_SOCC_ might lead to decreased Ca^2+^ oscillations in ASM cells. **(B)** The frequency of MCh (400 nM) induced Ca^2+^ oscillations in ASM cells of PCLS decreased when SOCE is inhibited by 1 μM and 10 μM GSK7975A (G7) respectively. **(C)** There are significant differences among Ca^2+^ oscillation frequency induced by 400 nM MCh, MCh +1 μM G7 and MCh +10 μM G7. Data are presented as the mean ± SD. For each bar in (C), seven to eight ASM cells obtained from 6 PCLS in 3 BALB/c mice are evaluated.

As SOCE is required for sustained agonist-induced Ca^2+^ oscillations in ASM (Chen and Sanderson, 2017), an inherently higher SOC current might contribute to the increased Ca^2+^ oscillations and thus stronger ASM contraction observed in BALB/c mice. Accordingly, we used an established mathematical model to predict the potential contribution of SOC current to the agonist-induced Ca^2+^ oscillations in ASM cells. As Figure 3A showed, the mathematical model predicted that the decreased magnitude of SOC current (V_SOCC_) reduced the frequency of Ca^2+^ oscillation. This prediction was validated in experiment that the blockage of SOCE by 1 μM and 10 μM GSK7975A, a specific inhibitor targets SCOE (Chen and Sanderson, 2017), significantly reduced MCh-induced Ca^2+^ oscillation frequency in a dose-dependent way in ASM cells of PCLS (Figures 3B,C). Therefore, both the mathematical model and experimental results suggested that SOCE magnitude regulates the Ca^2+^ oscillations frequency in ASM cells.

### 3.4 BALB/c mice showed higher [Ca^2+^]_i_ in SOCE-activated ASM cells, which might be SOC current-dependent

**FIGURE 4:**
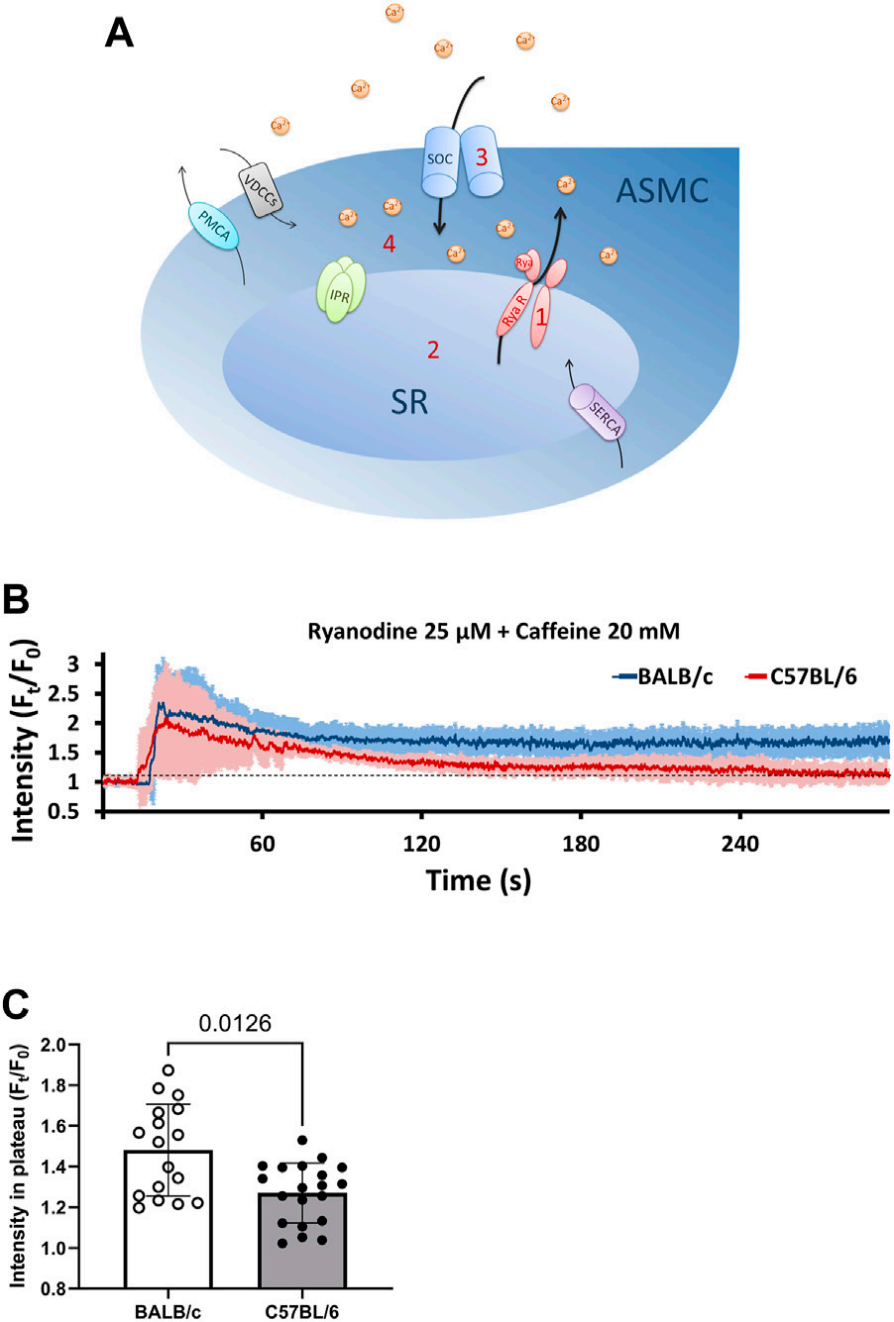
BALB/c mice show higher levels of [Ca^2+^]_i_ in SOCE-activated ASM cells than C57BL/6 mice. **(A)** A sketch illustrates the development of SOCE-activated ASM cells: **1**, PCLS were treated with 25 μM ryanodine (Rya) and 20 mM caffeine simultaneously to “lock” the ryanodine receptors (RyR) of SR in an open state; **2**, Opening of RyR leads to the depletion of SR Ca^2+^; **3**, Ca^2+^ depletion in SR induces Ca^2+^ influx through SOC to form SOCE; **4,** The steady level of [Ca^2+^]_i_ in SOCE-activated ASM cells results from a dynamic balance of Ca^2+^ influx through SOCE, Ca^2+^ efflux from SR through RyR, Ca^2+^ uptake to the SR by SERCA, and Ca^2+^ efflux through plasma membrane Ca^2+^ ATPase (PCMA) and Na^+^/Ca^2+^ exchanger (NCX). **(B)** SOCE-activated PCLS is prepared by exposing PCLS to a cocktail of 25 μM ryanodine and 20 mM caffeine at 37°C, ASM cells from BALB/c mice (BLUE line) show a higher steady level of [Ca^2+^]_i_ than those from C57BL/6 mice (RED line). Each point of the line represents the mean ± SD of the intensity at selected time points (F_t_) normalized to the initial intensity at the beginning time (F_0_). **(C)** Comparison of steady levels (F_t_/F_0_) in SOCE-activated ASM cells derived from BALB/c and C57BL/6 mice. Data are presented as the mean ± SD, *p* = 0.0126. Data are obtained from 17 ASM cells of 5 BALB/c mice, and 20 ASM cells of 5 C57BL/6 mice, respectively.

To further investigate the possible increased magnitude of SOCE in ASM cells of BALB/c mice, we utilized a model called “SOCE-activated PCLS”. The rationale of SOCE-activated PCLS has been described previously (Bai and Sanderson, 2006; Chen and Sanderson, 2017). Briefly, PCLS is treated with ryanodine and caffeine simultaneously. While caffeine stimulates RyR opening, ryanodine irreversibly locks the RyR in a submaximal open state, which results in constitutive Ca^2+^ efflux from SR and consequently decreased [Ca^2+^]_SR_. This activates and maintains the SOCE to induce Ca^2+^ influx (Figure 4A). The treatment first induces an immediate transient increase of [Ca^2+^]_i_ caused by Ca^2+^ released from the SR through RyR opening, followed by a sustained elevation of [Ca^2+^]_i_ (Figure 4B). The steady level of this elevated [Ca^2+^]_i_ results from a dynamic balance of Ca^2+^ influx through SOCE (SOC current, P_SOC_), Ca^2+^ efflux from SR through RyR (P_RyR_), Ca^2+^ uptake to the SR by SERCA (P_SERCA_), and intercellular Ca^2+^ efflux through plasma membrane Ca^2+^ ATPase (PCMA) and Na^+^/Ca^2+^ exchanger (NCX) (P_PCMA_ and P_NCX_, Figure 4A). As previously validated (Bai and Sanderson, 2006), the SOCE-activated PCLS presents an irreversible decrease of [Ca^2+^]_SR_ due to the constant opening of RyR and thus sustained activation of SOCE for at least 5 h since treatment of ryanodine and caffeine.

We used the mathematical model to predict the contribution of P_RyR_, P_SOC_ and P_SERCA_ to the steady level of elevated [Ca^2+^]_i_ in SOCE-activated ASM cells. As Supplementary Figure S3A, B show, with either a given P_RyR_ or a given P_SERCA_, the steady level of [Ca^2+^]_i_ changes little as P_SERCA_ or P_RyR_ increased. On the other hand, with a given P_RyR_, increased P_SOC_ leads to an increased [Ca^2+^]_i_ (Supplementary Figure S3C), indicating that the steady level of [Ca^2+^]_i_ is mainly determined by the P_SOC_ in SOCE-activated ASM cells. Interestingly, the influence of P_RyR_ on the steady level of [Ca^2+^]_i_ is only evidenced when the P_SOC_ is sufficient (Supplementary Figure S3D), indicating that the contribution of P_RyR_ to [Ca^2+^]i also depends on P_SOC_.

Accordantly, our experimental results showed that when exposed to the same [Ca^2+^]_e_, the steady level of [Ca^2+^]_i_ in SOCE-activated ASM cells derived from BALB/c PCLS was significantly higher than that observed in C57BL/6 PCLS (Figures 4B,C). This observation suggested a completely different dynamic balance of Ca^2+^ influx and efflux in SOCE-activated ASM cells between BALB/c and C57BL/6 mice. As this steady level of [Ca^2+^]_i_ is mainly determined by P_SOC_, it is assumed that the magnitude of SOCE in ASM cells of BALB/c mice is higher than those of C57BL/6 mice.

### 3.5 BALB/c mice showed higher SOC current in ASM cells than C57BL/6 mice

**FIGURE 5:**
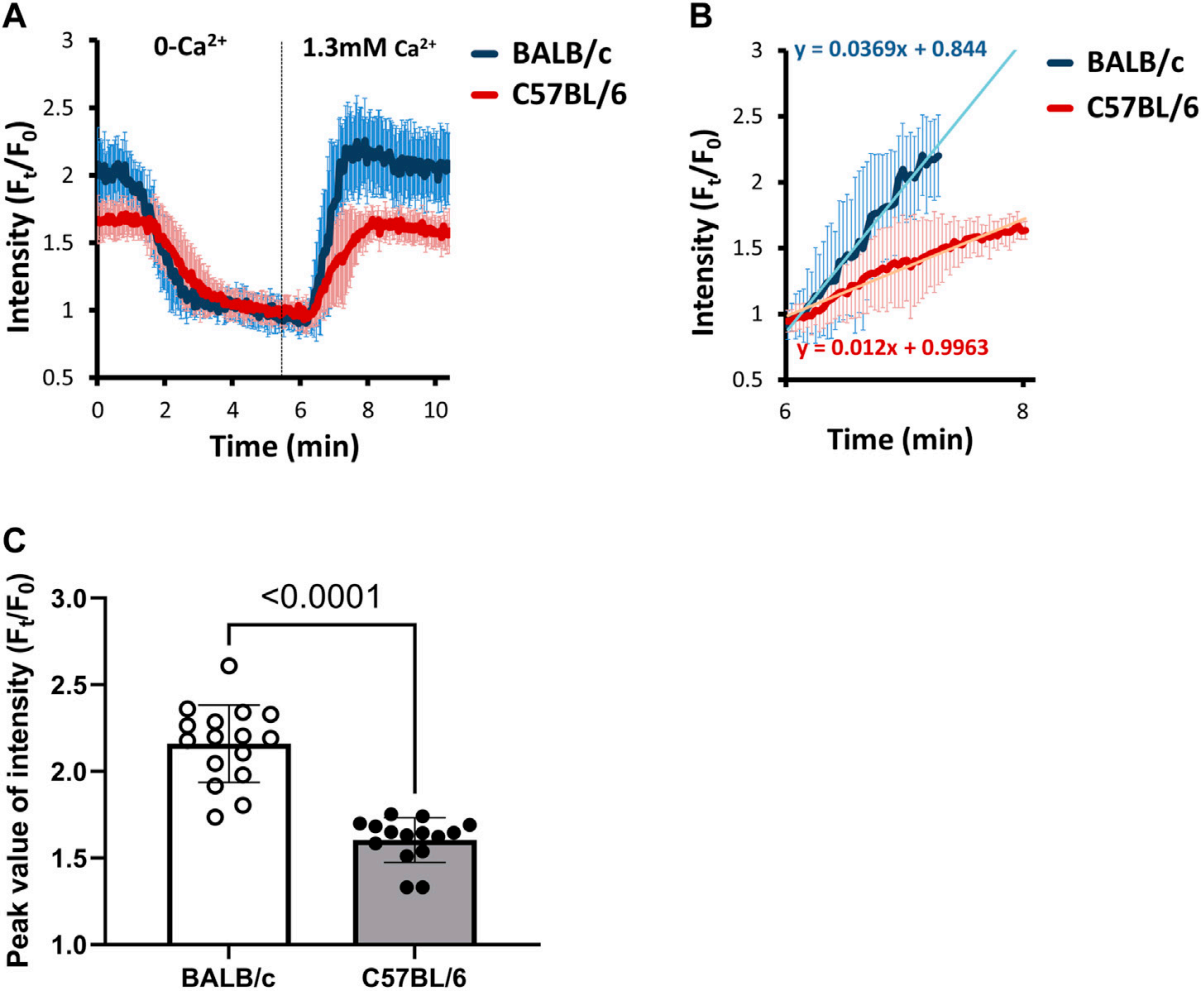
BALB/c mice show faster and higher SOC currents in SOCE-activated ASM cells than C57BL/6 mice. **(A)** The SOC current is evaluated by removing extracellular Ca^2+^ first to empty the [Ca^2+^]_i_ and [Ca^2+^]_SR_ in SOCE-activated ASM cells, and then adding back 1.3 mM Ca^2+^ to assess the transient Ca^2+^ influx via SOCE. Each point of the line represents the mean ± SD of the intensity at selected time points (F_t_) normalized to the initial intensity at the beginning time (F_0_). The SOC current was further quantified and compared by measuring **(B)** the rate of Ca^2+^ entry and **(C)** the peak value of Ca^2+^ influx. Data are presented as the mean ± SD, *p* < 0.0001 in **(C)**. Data are obtained from 16 ASM cells in 8 PCLS of 5 BALB/c mice, and 15 ASM cells in 7 PCLS of 5 C57BL/6 mice.

To further evaluate the SOC current in ASM cells, we removed the extracellular Ca^2+^ (by giving 0-Ca^2+^ sHBSS) to the SOCE-activated ASM cells firstly to decrease the [Ca^2+^]_i_ and [Ca^2+^]_SR_. As shown in Figure 5A, 0-Ca^2+^ sHBSS induced decreased [Ca^2+^]_i_ within 5 min. As previously described, the [Ca^2+^]_i_ is primarily determined by the SOC influx in the SOCE-activated ASM cells, we then added back 1.3 mM Ca^2+^ to assess the transient Ca^2+^ influx via SOCE (Figure 5A). The SOC current was quantified and compared by measuring the rate of Ca^2+^ entry (Figure 5B) and the peak value of Ca^2+^ influx (Figure 5C). The results showed that both the rate of Ca^2+^ entry and the peak value of Ca^2+^ influx via SOCE were significantly higher in ASM cells of BALB/c mice than those of C57BL/6 mice, suggesting that the ASM cells of BALB/c mice have a higher SOC current.

### 3.6 BALB/c mice show increased expression of STIM1 and Orai1 in ASM cells of small airways compared to C57BL/6 mice

**FIGURE 6:**
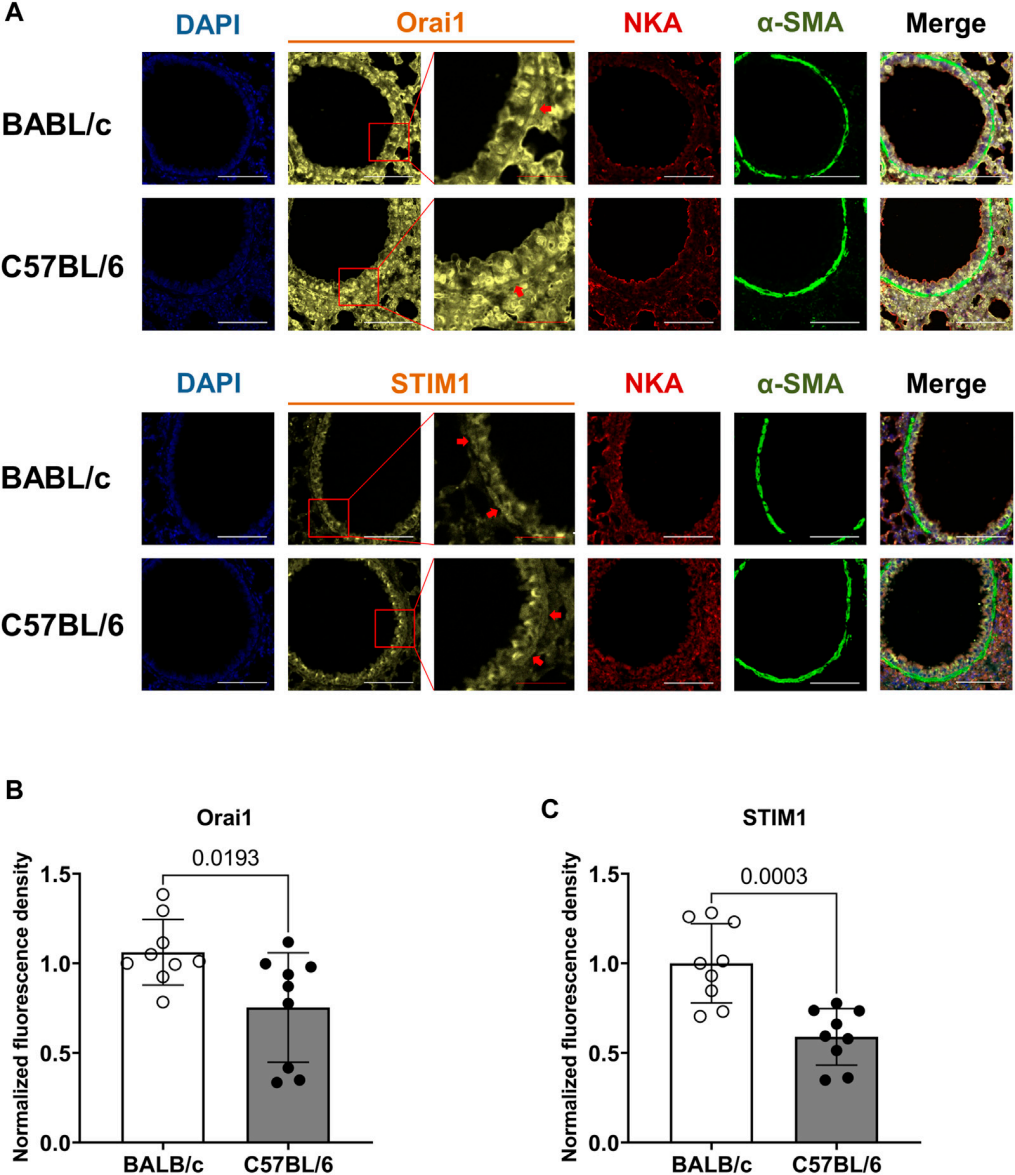
BALB/c mice show increased expression of Orai1 and STIM1, two essential component proteins of SOCE, in ASM cells of small airway compared to C57BL/6 mice. **(A)** The representative fluorescent images show the co-staining of DAPI, Orai1 or STIM1, Na^+^/K^+^ ATPase (NKA) and α-smooth muscle actin (α-SMA) in ASM cells of BALB/c and C57BL/6 mice; the fluorescent intensity of Orai1 or STIM1 were normalized to that of NKA in the same region of interest within ASM cells, which was identified by α-SMA; The red arrows indicate the representative ASM cells of interest used for analysis; White scale bar: 100 μm, red scale bar: 25 μm. **(B–C)** A comparison of the normalized fluorescent density of Orai1 (B) and STIM1 (C) in ASM cells derived from BALB/c and C57BL/6 mice, respectively. Data are presented as the mean ± SD, *p* = 0.0193 in (B), *p* = 0.0003 in (C). The bar in (B) represents 9 ASM cells obtained from 6 slices in three BALB/c mice, and 9 ASM cells obtained from 8 slices in three C57BL/6 mice; the bar in (C) represents 9 ASM cells obtained from 7 slices in three BALB/c mice, and 9 ASM cells obtained from 9 slices in three C57BL/6 mice.

To support a higher P_SOCE_ observed in ASM cells of BALB/c mice, we used semiquantitative immunofluorescent method to assess the expression of Orai1 and STIM1, two essential component proteins of SOCE, in the ASM cells of small airway derived from BALB/c and C57BL/6 mice. We used fluorescent intensity of NKA, a commonly used membrane marker, as the internal control to normalize the fluorescent intensity of Orai1 and STIM1 respectively (Figure 6A) in ASM cells labeled with α-smooth muscle actin (α-SMA). The results showed that the ASM cells of small airways obtained from BALB/c mice have higher intensity ratio of STIM1/NKA and Orai1/NKA respectively than those of C57BL/6 mice, indicating a relatively higher expression of essential elements of SOCE in ASM cells of BALB/c mice, in line with the larger SOC current observed in ASM cells of BALB/c mice.

## 4 Discussion

It is well known that airway responses appear differently among mouse strains, such as the contraction response to agonists (Levitt and Mitzner, 1988), IgE response to allergens (Larsen et al., 1992), force-velocity response in sensitized tracheal smooth muscle (TSM) strips (Fan et al., 1997), and different shortening velocities of ASM in both central and peripheral airways before or after sensitization with antigens (Duguet et al., 2000; Wagers et al., 2007). As BALB/c and C57BL/6 mice are widely used as animal models in studies of asthma and AHR, it is essential to characterize their innate differences in the airway response. Although BALB/c mice showed a higher agonist-induced airway response than C57BL/6 mice as assessed *in vivo* (Duguet et al., 2000), they showed no different response in the isometric tension of tracheal ring to agonists (Safholm et al., 2011). We hypothesized that the small airway contraction of BALB/c mice might differ from that of C57BL/6 mice, which could explain their different airway responses. Here, by using PCLS, a useful tool to investigate ASM physiology in relatively small airways *in situ*, we show that BALB/c mice have stronger extent of small airway narrowing and increased Ca^2+^ oscillation frequency in ASM cells in response to agonists than C57BL/6 mice. These observations are consistent with the increased airway response and ASM shortening velocity observed in BALB/c mice. As inspired by the increased steady level of [Ca^2+^]_i_ observed in SOCE-activated ASM cells derived from BALB/c mice, we further show that ASM cells derived from BALB/c mice have inherently higher Ca^2+^ influx via SOCE and increased expressions of Orai1 and STIM1 compared to those derived from C57BL/6 mice. Furthermore, both the mathematical model and the experimental results suggest that decreased SOC current leads to decreased frequency of Ca^2+^ oscillation in ASM cells. Therefore, the inherently higher SOC current resulting from increased expression of Orai1 and STIM1 might account for the higher frequency of Ca^2+^ oscillation and stronger contraction of ASM cells in BALB/c mice than in C57BL/6 mice.

Previous studies have demonstrated that the agonist-induced Ca^2+^ oscillations are generated by the cyclic release and re-uptake of Ca^2+^ from and to the SR (Wright et al., 2013). Specifically, agonist (such as MCh and 5-HT) binds to their respective cell-surface receptors and activates the different downstream G protein-coupled pathways to produce IP_3_, which binds IP_3_R to release Ca^2+^ from the SR into the cytoplasm; Ca^2+^ in the cytoplasm is pumped back into the SR by SERCA (Prakash et al., 2009). Ca^2+^ efflux from the SR might also occur via RyR (Croisier et al., 2015). During Ca^2+^ oscillations, the cytoplasmic Ca^2+^ continually loss via PMCA and NCX, therefore, an in-time supplementation of intracellular Ca^2+^ is essential for the sustained agonist-induced Ca^2+^ oscillations in ASM. We have demonstrated that SOCE, rather than voltage-gated Ca^2+^ channels, is the major Ca^2+^ influx pathway for sustaining agonist-induced Ca^2+^ oscillations in ASM cells (Chen and Sanderson, 2017).

The molecular basis of SOCE (Orai1 and STIM1) has been well demonstrated (Prakriya and Lewis, 2015). STIM1 senses the decreased [Ca^2+^]_SR_, and then oligomerizes and translocates to a region near the PM, where it interacts with Orai1 to generate a local Ca^2+^ influx current to replenish the Ca^2+^ lost in ASM cells (Croisier et al., 2013). Previous studies reported that the expressions of STIM1 and Orai1 are increased in ASM cells derived from experimental asthmatic mice (Spinelli et al., 2012; Qiu et al., 2017). In addition, STIM1 and Orai1-mediated SOCE has been associated with the proliferation of ASM cells (Zou et al., 2011). Furthermore, STIM1 is required for ASM remodeling through metabolic and transcriptional reprogramming and cytokine secretion (Johnson et al., 2022). In the present study, we demonstrate that inherently higher SOC current might induce increased Ca^2+^ oscillation frequency in ASM cell, leading to increase of [Ca^2+^]_i_ due to spatial and temporal integration, and thus resulting in stronger contraction. These results together highlight the regulatory role of SOCE in the Ca^2+^ oscillations of ASM cells and the contraction of ASM, which is closely associated with AHR.

It is worth noting that agonist-induced airway contraction is not only determined by the Ca^2+^ oscillations of ASM cells. Previously, we reported that both the Ca^2+^ oscillation frequency and the Ca^2+^ sensitivity of ASM cells contribute to innate ASM contractility (Bergner and Sanderson, 2003; Bai and Sanderson, 2009), whether the Ca^2+^ sensitivity of ASM cells in BALB/c mice differs from that in C57BL/6 mice is still unknown and worth further investigation. In addition, it is reported that different SERCA expression (Chen et al., 2017), IP_3_R receptor density (Narayanan et al., 2012) and imbalance of GPCR-derived signals (Wright et al., 2013) could affect the agonist-induced Ca^2+^ oscillation frequency, whether BALB/c and C57BL/6 mice have different SERCA or IP_3_R expression might need further investigation. Furthermore, it has been reported that the contractile protein content in ASM cells (Ammit et al., 2000), the tone of ASM cells themself (Fairbank et al., 2008), and the extracellular matrix stiffness (Polio et al., 2019) also exert impacts on airway contractility, however, whether these factors contribute to the different extents of small airway narrowing between BALB/c and C57BL/6 mice is unclear, but beyond our scope here. Finally, the absolute quantification of Orai1 and STIM1 protein expression in the ASM cells of small airway should be investigated in future study.

Taken together, our findings demonstrate that, as compared to C57BL/6 mice, BALB/c mice have inherently higher small airway contraction along with increased agonist-induced Ca^2+^ oscillation frequency, which might result from higher SOCE influx due to increased expression of Orai1 and STIM1 in ASM cells. These results suggest that an increased SOC current in ASM itself might lead to a higher Ca^2+^ oscillation frequency and stronger airway contraction, highlighting the importance of SOCE in understanding the mechanisms of AHR.

## Data Availability

The original contributions presented in the study are included in the article/Supplementary Material, further inquiries can be directed to the corresponding author.
