# Supplementary material for: Inherent differences of small airway contraction and Ca2+ oscillations in airway smooth muscle cells between BALB/c and C57BL/6 mouse strains
Source: Front Cell Dev Biol. 2023 Jun 5;11:1202573. doi: 10.3389/fcell.2023.1202573 (PMC10279852; doi:10.3389/fcell.2023.1202573)
Supplement: Supplementary file 1 [file Presentation1.PPTX]

## Slide 1
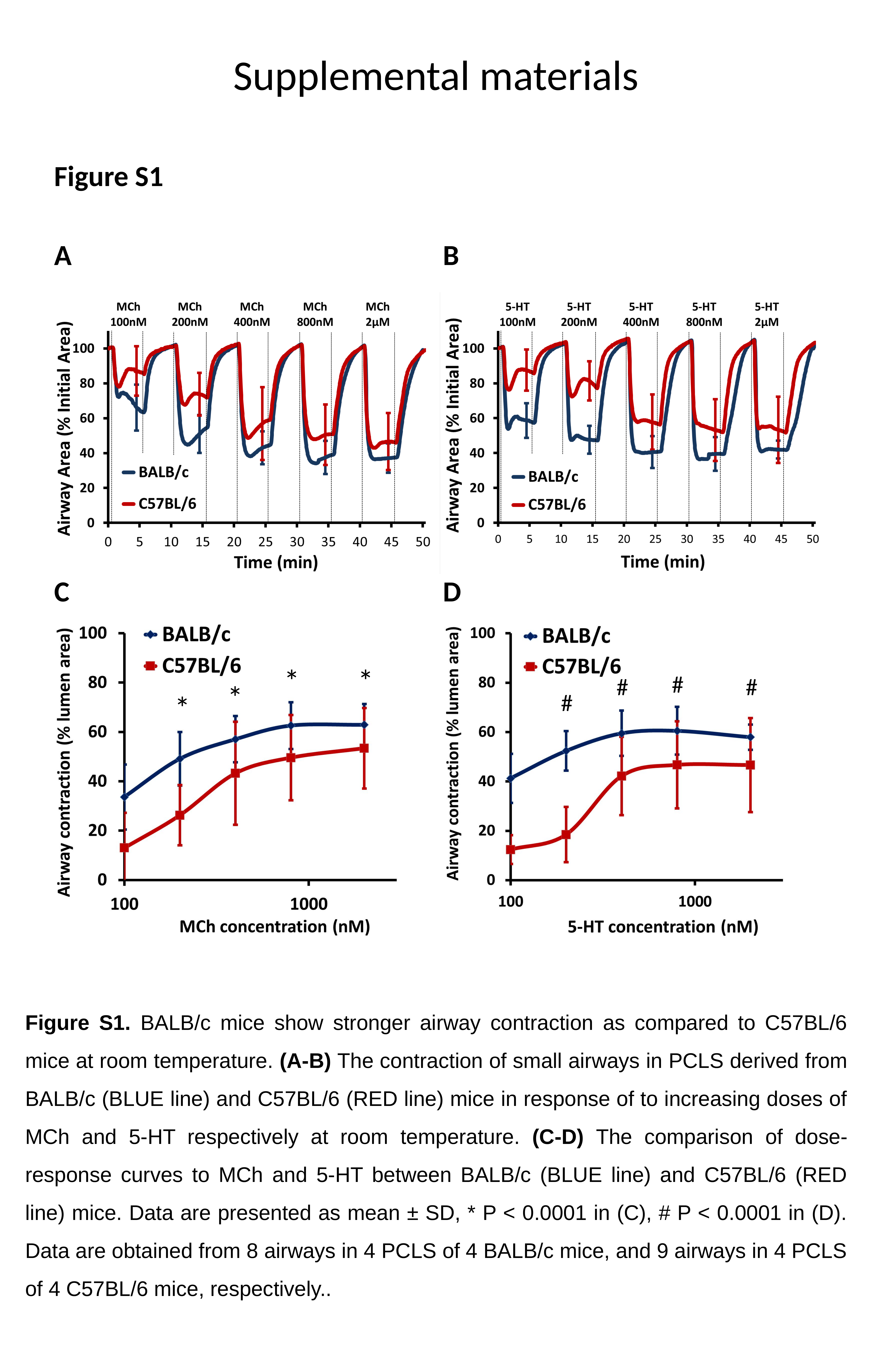

Supplemental materials
Figure S1
A
B
C
D
*
*
#
#
#
*
#
*
Figure S1. BALB/c mice show stronger airway contraction as compared to C57BL/6 mice at room temperature. (A-B) The contraction of small airways in PCLS derived from BALB/c (BLUE line) and C57BL/6 (RED line) mice in response of to increasing doses of MCh and 5-HT respectively at room temperature. (C-D) The comparison of dose-response curves to MCh and 5-HT between BALB/c (BLUE line) and C57BL/6 (RED line) mice. Data are presented as mean ± SD, * P < 0.0001 in (C), # P < 0.0001 in (D). Data are obtained from 8 airways in 4 PCLS of 4 BALB/c mice, and 9 airways in 4 PCLS of 4 C57BL/6 mice, respectively..

## Slide 2
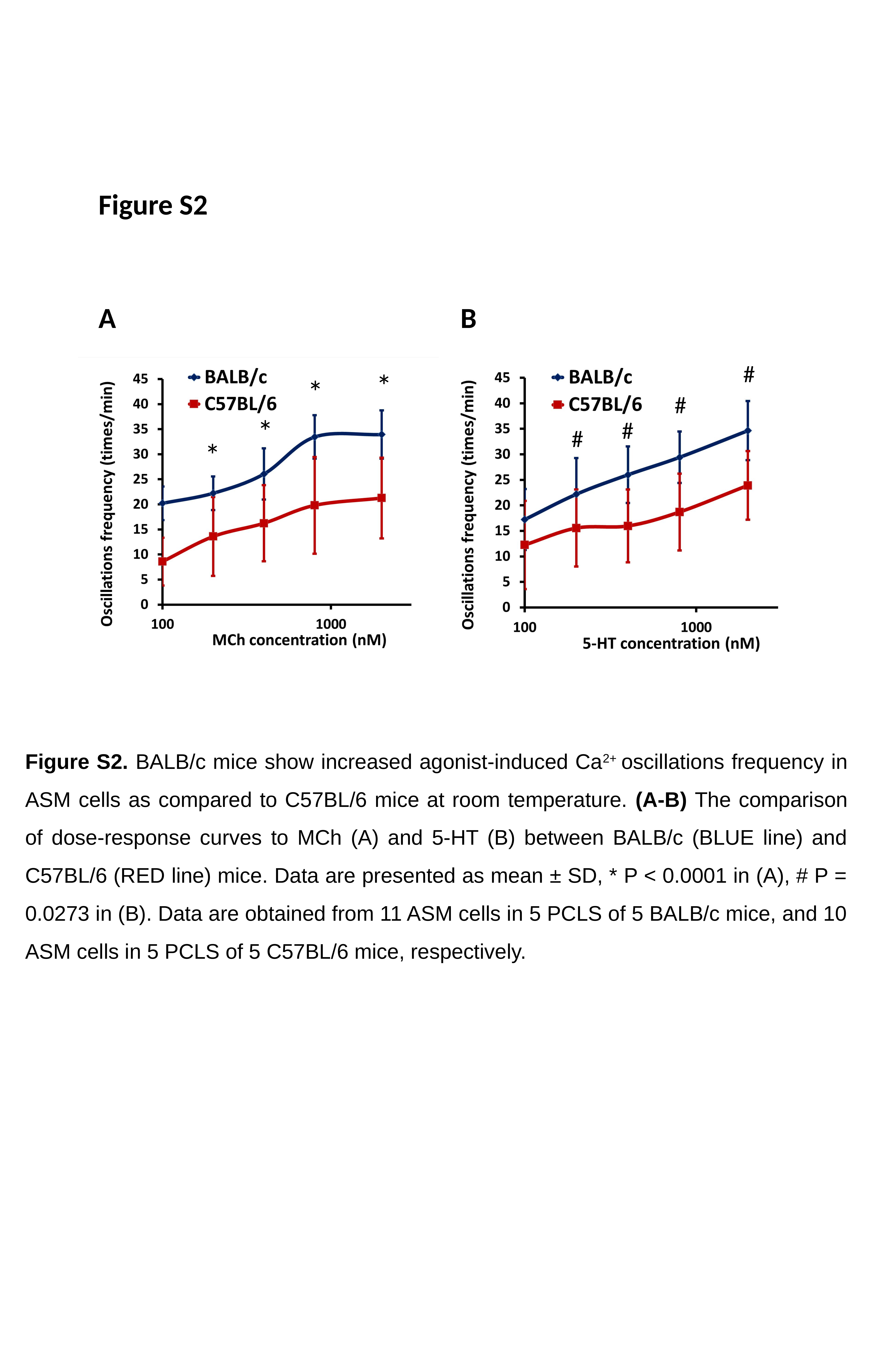

Figure S2
A
B
#
*
*
#
*
#
#
*
Figure S2. BALB/c mice show increased agonist-induced Ca2+ oscillations frequency in ASM cells as compared to C57BL/6 mice at room temperature. (A-B) The comparison of dose-response curves to MCh (A) and 5-HT (B) between BALB/c (BLUE line) and C57BL/6 (RED line) mice. Data are presented as mean ± SD, * P < 0.0001 in (A), # P = 0.0273 in (B). Data are obtained from 11 ASM cells in 5 PCLS of 5 BALB/c mice, and 10 ASM cells in 5 PCLS of 5 C57BL/6 mice, respectively.

## Slide 3
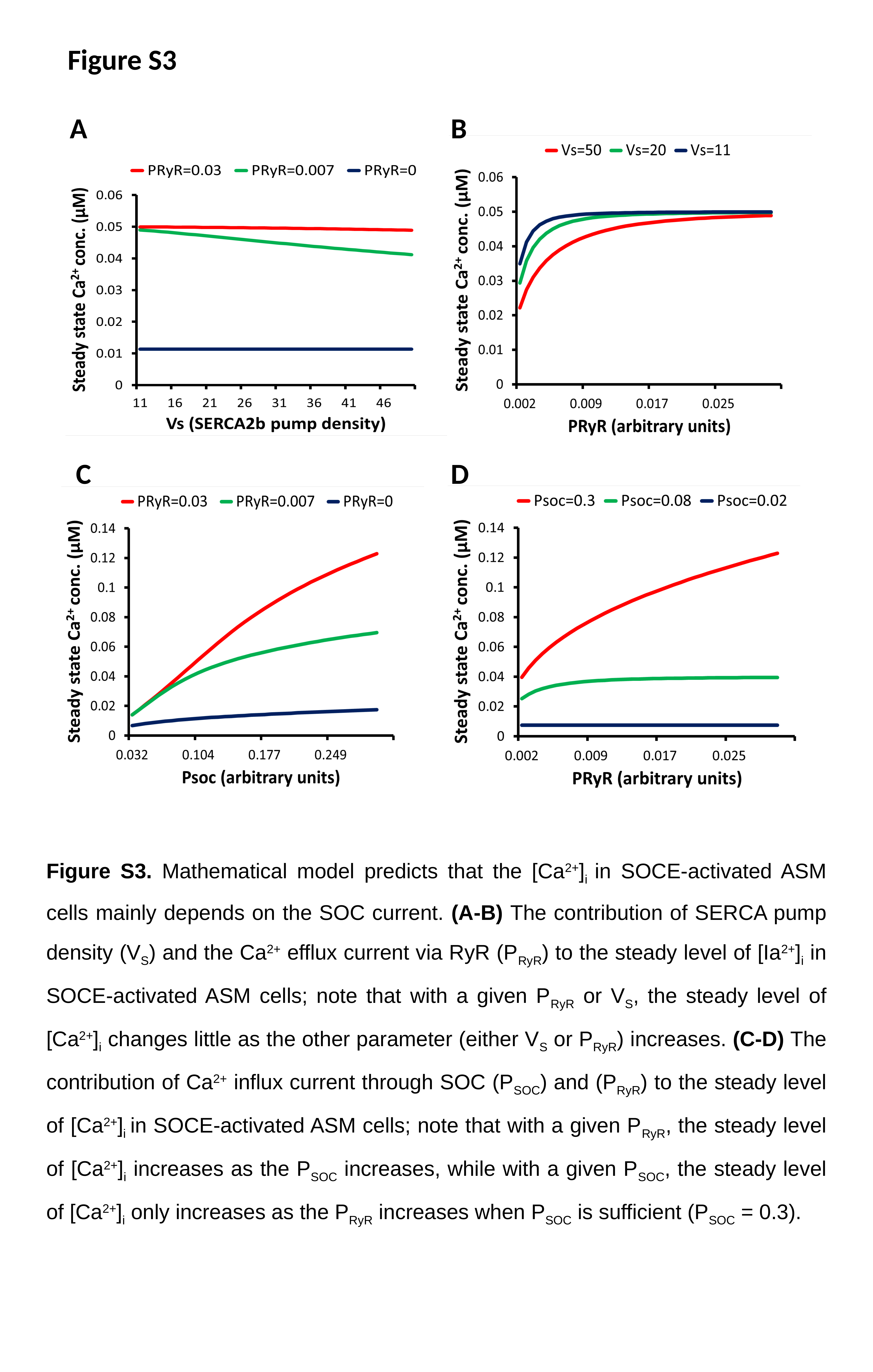

Figure S3
A
B
C
D
Figure S3. Mathematical model predicts that the [Ca2+]i in SOCE-activated ASM cells mainly depends on the SOC current. (A-B) The contribution of SERCA pump density (VS) and the Ca2+ efflux current via RyR (PRyR) to the steady level of [Ia2+]i in SOCE-activated ASM cells; note that with a given PRyR or VS, the steady level of [Ca2+]i changes little as the other parameter (either VS or PRyR) increases. (C-D) The contribution of Ca2+ influx current through SOC (PSOC) and (PRyR) to the steady level of [Ca2+]i in SOCE-activated ASM cells; note that with a given PRyR, the steady level of [Ca2+]i increases as the PSOC increases, while with a given PSOC, the steady level of [Ca2+]i only increases as the PRyR increases when PSOC is sufficient (PSOC = 0.3).
